# Initial response of ovarian tissue transcriptome to vitrification or microwave-assisted dehydration in the domestic cat model

**DOI:** 10.1186/s12864-020-07236-z

**Published:** 2020-11-25

**Authors:** Olga Amelkina, Pierre Comizzoli

**Affiliations:** Smithsonian Conservation Biology Institute, National Zoological Park, Washington, DC USA

**Keywords:** Vitrification, Dehydration, Ovarian cortex, Domestic cat, RNA-seq, Networks

## Abstract

**Background:**

Long term preservation of living ovarian tissues is a critical approach in human reproductive medicine as well as in the conservation of rare animal genotypes. Compared to single cell preservation, optimization of protocols for tissues is highly complex because of the diversity of cells responding differently to non-physiological conditions. Using the prepubertal domestic cat as a model, the objective was to study immediate effects of vitrification or microwave-assisted dehydration on the global transcriptome dynamics in the ovarian cortex. RNA sequencing was performed on ovarian tissues (*n* = 6 individuals) from different conditions: fresh tissue after dissection (F), vitrified/warmed tissue (V), tissue dehydrated for 5 min (D5) or 10 min (D10) followed by rehydration. Differential gene expression analysis was performed for comparison pairs V vs. F, D10 vs. F, D5 vs. F and D10 vs. D5, and networks were built based on results of functional enrichment and in silico protein-protein interactions.

**Results:**

The impact of the vitrification protocol was already measurable within 20 min after warming and involved upregulation of the expression of seven mitochondrial DNA genes related to mitochondrial respiration. The analysis of D10 vs. F revealed, 30 min after rehydration, major downregulation of gene expression with enrichment of in silico interacting genes in Ras, Rap1, PI3K-Akt and MAPK signaling pathways. However, comparison of D5 vs. F showed negligible effects of the shorter dehydration protocol with two genes enriched in Ras signaling. Comparison of D10 vs. D5 showed downregulation of only seven genes. Vitrification and dehydration protocols mainly changed the expression of different genes and functional terms, but some of the differentially expressed genes formed a major in silico protein-protein interaction cluster enriched for mitochondrial respiration and Ras/MAPK signaling pathways.

**Conclusions:**

Our results showed, for the first time, different effects of vitrification and microwave-assisted dehydration protocols on the global transcriptome of the ovarian cortex (using the domestic cat as a biomedical model). Acquired data and networks built on the basis of differentially expressed genes (1) can help to better understand stress responses to non-physiological stresses and (2) can be used as directions for future preservation protocol optimizations.

**Supplementary Information:**

The online version contains supplementary material available at 10.1186/s12864-020-07236-z.

## Background

The ovarian tissue is an untapped source of early germ cells that can be potentially used for fertility preservation. Techniques to safeguard the cortical pool of preantral follicles have been constantly under development [1]. Ovarian cortical preservation can potentially be performed at any age or reproductive status of females, making it a valuable biomaterial in biomedical science [2] and in conservation of rare and endangered genotypes [3]. So far, two methods of ovarian tissue preservation have been extensively developed: slow-freezing and vitrification [4]. During slow freezing, there are more chances of ice nucleation followed by damaging crystal growth when the molecular arrangement of water is altered to a solid pattern [5]. However, vitrification technique avoids this by relying on rapid drop in temperature with no time or energy for molecular rearrangement. The maintenance of natural disorder of liquid molecules in tissue and mitigation of any disturbance to the system leads to a stable glass formation. The procedure of vitrification has been optimized by increasing the viscosity of the solution, which prevents the crystal formation and therefore avoids cellular damage [5]. However, the increase in viscosity requires high concentrations of cryoprotective agents (CPAs) in order to extract water from the tissue, creating possible toxic effects of CPAs on the cells [6]. Protocols have been optimized to avoid this issue, e.g., by adjusting the concentration of CPAs or by exposing samples to low temperature before vitrification to decrease the rate of potential energy and cell metabolism, making molecules less mobile and more stagnant but still disordered [7, 8].

Alternative methods of cell preservation that avoid the reliance on low temperatures have been under investigation, including cell dehydration and storage at ambient temperatures. The idea of preserving cells in dry state comes from organisms called anhydrobiotes that survive environmentally driven huge losses of (> 95%) intracellular water [9]. Through extensive studies it was learned that anhydrobiotes use disaccharides (mainly trehalose) and hydrophilins (small intrinsically disordered proteins) to mitigate desiccation-induced cellular damage [10–14]. Without protective factors, desiccation can impose a number of stresses, including hyperosmolarity, hyperoxidation, hyperionicity, and protein misfolding/aggregation, all of which have the potential to inflict lethal damage upon DNA, proteins, and membranes [15]. Studies showed that trehalose can vitrify to prevent erroneous protein-protein interactions that lead to protein unfolding and aggregation [16]; it can also associate with membranes to help protect them during water removal, thereby eliminating the detrimental solid-liquid transition upon rehydration [11].

Our laboratory has been developing both vitrification and alternative preservation methods for gametes and gonads using the domestic cat as a model for humans and for rare and endangered felids due to comparable traits in ovarian anatomy and physiology [17, 18]. Prepubertal ovarian tissues have been used due to their high reserves of primordial and primary follicles and homogeneity compared to adult counterparts [19]. Our vitrification protocol has been optimized so there is no significant differences in percentage of morphologically normal follicles between vitrified/warmed and fresh cortical tissue, and only mild decrease in follicular viability and RNA synthesis [8, 20]. Our laboratory also reported initial steps in the development of microwave-assisted dehydration technique for cat germinal vesicle [21, 22], sperm cells [23], and most recently ovarian tissue [18], relying on intracellular delivery of trehalose as protection from dehydration/rehydration damage. After cortical tissue dehydration for up to 30 min followed by immediate rehydration, there was no significant change in the percentage of morphologically normal follicles or stromal cell density, and no significant increase in DNA damage; however, there was a significant decrease in RNA synthesis in follicles already after 10 min of drying [18].

Optimization of cell preservation techniques requires deep understanding of the processes happening in the cell in response to preservation. Several studies have used RNA sequencing (RNA-seq) to measure global transcriptomic changes in oocytes after vitrification followed by culturing or in vitro maturation [24–28], which provided informative transcriptomic data on the oocyte state after at least several hours post vitrification/warming. Another recent study performed RNA-seq on mouse ovarian tissue after vitrification followed by 20 days of autotransplantation [29]. It appears essential to investigate cellular transcriptomic changes immediately after completing the preservation/reanimation to track the initial cellular response to potential stresses caused by specific techniques.

Analyzing transcription dynamics from various stresses can help elucidate the mechanisms that prepare and execute genome-wide changes in gene expression in particular cells and tissues [30]. Physiological responses in the eukaryotic organism typically involve coordinated actions of many cells as the roles in maintaining homeostasis is divided among different cell types. Therefore, cell communication is potentially as important as the cell-autonomous response to physiological stimuli in animals, and the diversity of distinct differentiated cell types may be accompanied by a corresponding diversity in their responses to stress [31]. The ovarian cortex is a specialized tissue with intricate system involving several types of cells; recent study using single-cell RNA-seq identified six main cell populations in the human ovarian cortex: oocytes, immune cells, endothelial cells, granulosa cells, perivascular cells and stroma [32]. Additionally, different types of granulosa and theca cells were detected using single-cell RNA-seq in the inner part of human ovaries [33]. It is therefore important to measure a whole ovarian tissue transcriptomic response to potential stresses caused by preservation protocols to make a progress in developing and adapting various ovarian cortex preservation techniques.

Using the prepubertal cat model, the objective was to analyze the transcription dynamics in response to stress caused by vitrification or dehydration in the ovarian cortex. Specifically, RNA-seq was performed on groups representing different state of ovarian tissue: fresh tissue after ovary dissection, tissue subjected to vitrification and warming, tissue subjected to microwave-assisted dehydration and rehydration. A global transcriptomic approach was chosen to identify first and foremost affected functional terms and pathways, to have a clearer overview of potential global effects of vitrification and dehydration. Observations were made at the same time points after warming or rehydration as in our previous studies to be able to relate new transcriptomic data to earlier findings.

## Results

### Summary of the acquired dataset

**Fig. 1:**
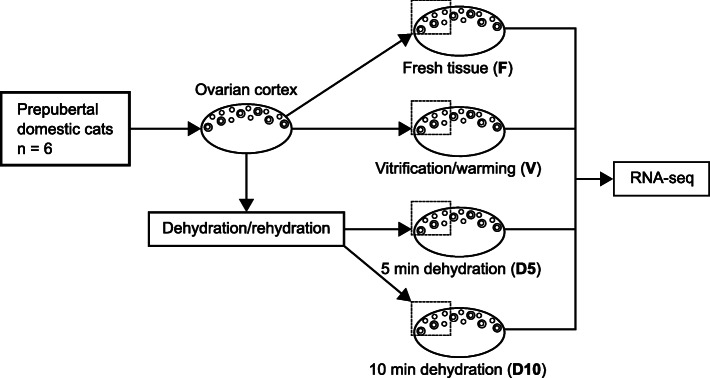
Experimental design

Ovaries from six prepubertal domestic cats were each divided into four groups: fresh tissue after ovary dissection (F), tissue subjected to vitrification and warming (V), tissue subjected to microwave-assisted dehydration for 5 min (D5) or 10 min (D10) with subsequent rehydration (Fig. 1). Within 30 min post-warming or rehydration, samples were stabilized in RNA-later for RNA-seq. Whole transcriptome of the resulting 24 samples was sequenced with one library per each sample, six biological replicates per group, 30 million read depth per sample and 150 bp paired-end read length. The acquired sequence data in fastq format is deposited to NCBI Sequence Read Archive; BioProject accession number is PRJNA662384. Data retrieved after differential gene expression analysis is presented in xlsx format for comparison pairs V vs. F (Additional file 1) and D10 vs. F, D5 vs. F and D10 vs. D5 (Additional file 2); Additional file 3 contains united differential expression data for all comparison pairs. Data used to create networks is available in the interactive web session view for each network (see below).

### Vitrification mainly upregulates the expression of genes involved in mitochondrial respiration

**Fig. 2:**
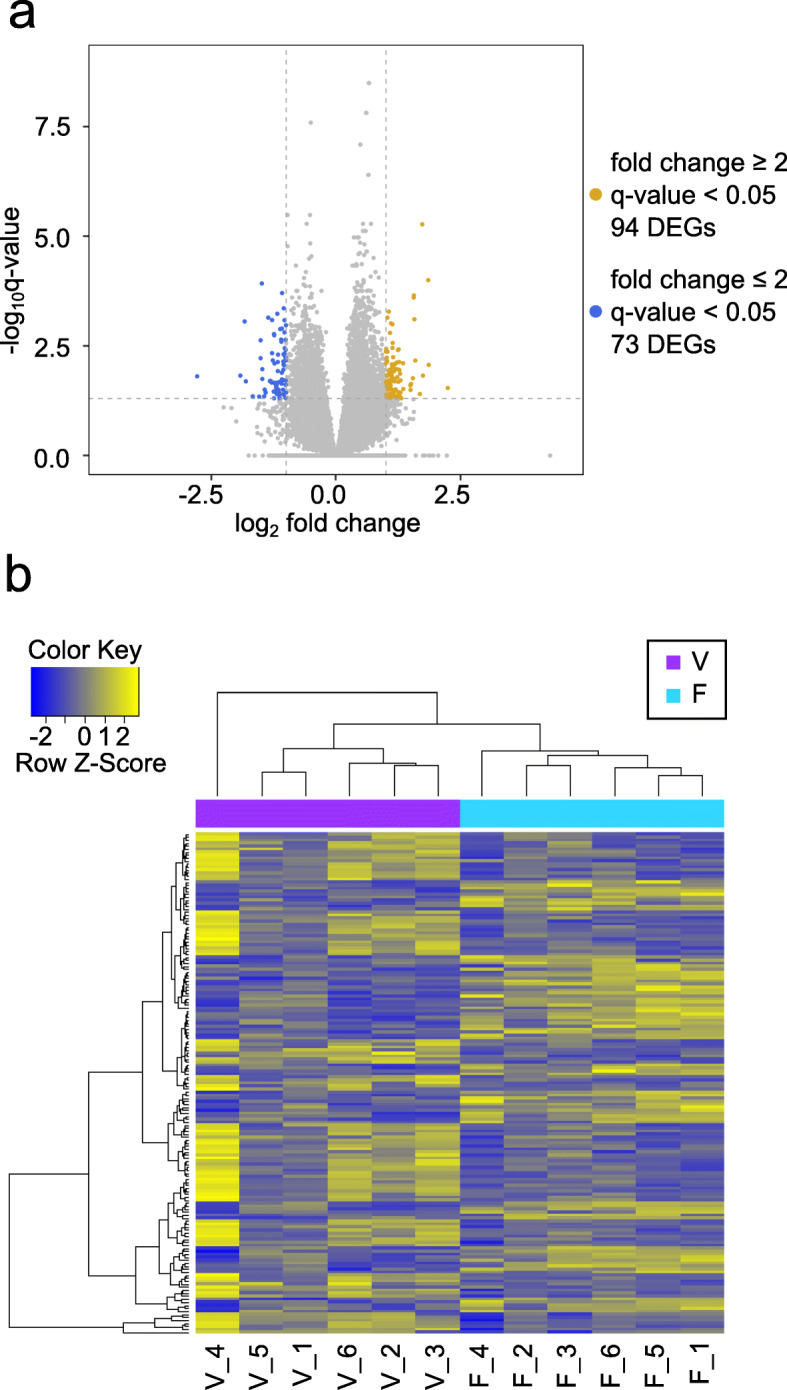
Influence of vitrification on the gene expression in cat ovarian cortex. **a** Volcano plot showing differentially expressed genes (DEGs) meeting the conditions of |fold change| ≥ 2 and q-value < 0.05. Yellow dots represent genes up-regulated in vitrification (V) vs. fresh tissues (F), blue dots represent genes down-regulated in V vs. F, and grey dots represent the genes that were not differentially expressed in V vs. F. **b** Heatmap of the one-way Hierarchical Clustering Analysis (Euclidean Method, Complete Linkage) using Z-score for normalized value (log_2_ based) for comparison pair V vs. F. Bottom of heatmap is labeled with sample IDs representing condition (V, F) and animal (1 to 6)

One hundred sixty seven genes were differentially expressed in the ovarian cortex after vitrification and warming (comparison pair V vs. F; Fig. 2a, Additional file 1). Of these, 94 were upregulated and included 59 protein coding genes, 2 pseudogenes, 29 lncRNAs, 2 rRNA sand 2 tRNAs. Seventy three genes were downregulated and included 44 protein coding genes, 4 pseudogenes and 25 lncRNAs. Hierarchical clustering analysis on resulted differentially expressed genes (DEGs) clearly divided samples into two clusters based on the treatment group (Fig. 2b).

**Fig. 3:**
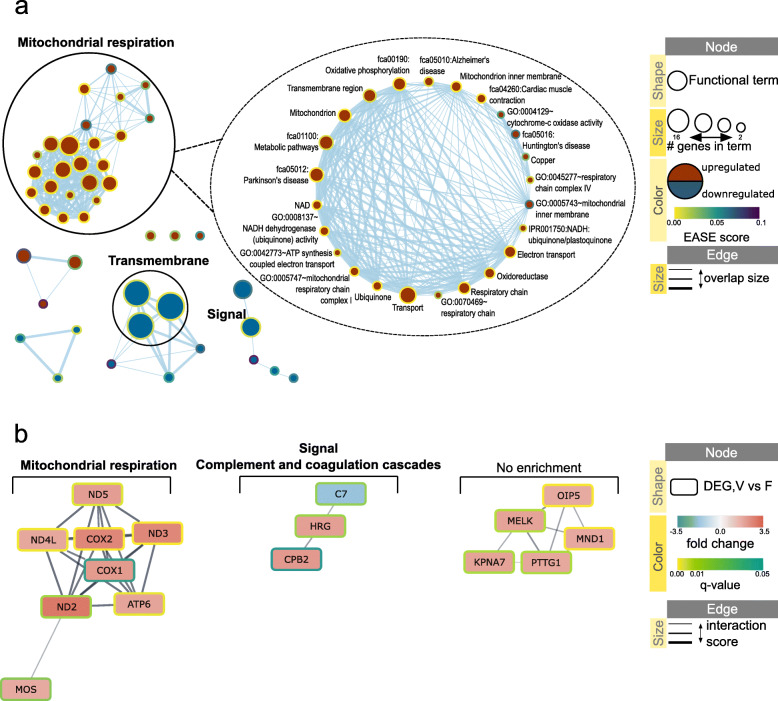
Network visualization of the effect of vitrification/warming on cat ovarian cortex. **a** Enrichment map displaying enriched gene-sets based on differentially expressed genes between vitrified (V) and fresh (F) tissue. Red node color represents enrichment in vitrified ovarian cortex (i.e., up-regulation after vitrification), whereas blue represents enrichment in fresh tissue (i.e., down-regulation after vitrification). Borders are colored according to the enrichment *p*-value (EASE score). Clusters of functionally related gene-sets were identified using DAVID tool, manually circled and assigned a label. Additional file 4 contains the web session of the network for interactive view. **b** STRING network for 89 proteins retrieved from the list of differentially expressed genes in comparison pair V vs. F. Full gene list of the pair V vs. F resulted in 38 functional associations between 18 of the proteins; proteins with less than 2 associations to other proteins in the network were removed from visualization. Each cluster was analyzed for functional enrichment within STRING app. Nodes are colored according to the gene abundance (fold change) in comparison pair V vs. F; borders are colored according to the adjusted *p*-value (q-value) of the differentially expressed gene. The confidence cutoff was set to 0.4, the confidence score of each interaction is mapped to the edge thickness. Additional file 5 contains the web session of the network for interactive view

Out of 103 protein coding DEGs, 102 were annotated in DAVID database; functional enrichment analysis was performed separately for upregulated (59) and downregulated (43) annotated DEGs. DAVID annotation clustering tool was used to create clusters of functionally related terms; information for each resulting annotation cluster is presented in Table 1, including results from sections below. Figure 3a visualizes DAVID results using Enrichment Map app in Cytoscape with mapped EASE scores (modified Fisher Exact *p*-value of enrichment). Additional file 4 contains the web session of the network with interactive view and data table.

**Table 1:** Enrichment scores for DAVID annotation clusters

Annotation cluster	Enrichment score	#genes	#terms	DEG source	DEG regulation	Visualization
Mitochondrial respiration	4.50	13	17	V vs F	up	Figure 3a
Transmembrane	2.07	16	3	V vs F	down	Figure 3a
Fibronectin type III	4.15	20	3	D10 vs F	down	Figure 5a
Transmembrane	3.73	56	4	D10 vs F	down	Figure 5a
Immunoglobulin-like domain	3.16	19	8	D10 vs F	down	Figure 5a
Ras signaling	2.34	17	6	D10 vs F	down	Figure 5a
IPT	2.03	6	3	D10 vs F	down	Figure 5a
RNA polymerase II activity	1.51	10	4	D10 vs F	down	Figure 5a
Transcription regulation, RNA polymerase II	1.18	12	4	D10 vs F	down	Figure 5a
Semaphorin	1.42	5	7	D10 vs F	down	Figure 5a
Cadherin	1.32	5	5	D10 vs F	down	Figure 5a
Transmembrane	1.76	32	4	V vs F	up&down	Figure 6a
Transmembrane	4.28	58	4	D10 vs F	up&down	Figure 6a
Leucine-rich repeat	1.18	5	6	D10 vs F	up&down	Figure 6a

Enrichment score**:** ranks the biological significance of gene groups based on overall EASE scores of all enriched annotation terms. Higher number indicates higher biological significance of the cluster

*IPT* Immunoglobulin-like, plexins and transcription factors

*F* Fresh ovarian tissue after dissection, *V* Vitrified/warmed tissue, *D10* Tissue dehydrated for 10 min and rehydrated

STRING database then was used to create the network of protein-protein interactions based on 89 out of 103 protein coding DEGs present in domestic cat database. For each cluster of genes that had at least two direct protein interactions, functional enrichment analysis in STRING app was performed. Thus, it was possible to consider pairwise relationships among interacting genes when checking for biological significance. Results of functional enrichment are presented in Table 2, including results from sections below. Figure 3b visualizes STRING results using STRING app in Cytoscape with mapped fold change and q-value of DEGs. Additional file 5 contains the web session with interactive view and data table.

**Table 2:** Enrichment analysis of directly interacting genes using STRING

PPI cluster	PPI enrichment	#genes in cluster	#enriched terms	DEG source	DEG regulation	Visualization
Mitochondrial respiration	1.0E-16	8	39	V vs F	up	Figure 3b
Signal, Complement and coagulation cascades	1.04E-4	3	2	V vs F	up&down	Figure 3b
Ras, Rap1, PI3K-Akt and MAPK signaling pathways +	1.0E-16	36	74	D10 vs F	up&down	Figure 5b
Calcium-binding EGF-like domain	5.95E-09	4	9	D10 vs F	down	Figure 5b
Ras, Rap1, PI3K-Akt and MAPK signaling pathways, Signal, Complement and coagulation cascades +	1.0E-16	48	74	V vs F, D10 vs F	up&down	Figure 6b
Transmembrane helix	1.8E-8	4	6	V vs F, D10 vs F	up&down	Figure 6b
Leucine-rich repeat	1.64E-09	5	2	V vs F, D10 vs F	up&down	Figure 6b

PPI enrichment**:** FDR adjusted *p*-value of cluster functional enrichment

*F* Fresh ovarian tissue after dissection, *V* Vitrified/thawed tissue, *D10* Tissue dehydrated for 10 min and rehydrated

Mitochondrial respiration DAVID cluster included terms with highest EASE score and had the highest cluster enrichment score (Fig. 3a, Table 1). All genes in this cluster were upregulated (Fig. 3a); 7 of these genes formed a direct protein interaction cluster in STRING network with retrieved functional enrichment in mitochondrial respiration and included mitochondrial DNA genes ND2, ND3, ND4L, ND5, COX1, COX2 and ATP6 (Fig. 3b, Table 2). In mitochondrial respiration protein interaction cluster, MOS directly interacted with ND2 (Fig. 3b); this gene was present in three enriched terms retrieved from DAVID analysis: ‘fca04114:Oocyte meiosis’, ‘ATP-binding’ and ‘Nucleotide-binding’ (Additional file 4).

Transmembrane DAVID cluster included 16 downregulated DEGs (Table 1) that did not form any interaction cluster in STRING network. Functional term ‘Signal’ was highly enriched (Fig. 3a), and one downregulated DEG from this term (C7) together with two other upregulated DEGs (HRG, CPB2), formed a protein interaction cluster with enrichment in ‘Signal’ and ‘Complement and coagulation cascades’ (Fig. 3b, Table 2).

In sum, the most significant biological effect of the vitrification protocol on ovarian cortex transcriptome was measurable within 20 min post-warming and involved upregulation of the expression of mitochondrial DNA genes related to mitochondrial respiration.

### Dehydration downregulates the expression of genes involved in Ras, Rap1, PI3K-Akt and MAPK signaling pathways

**Fig. 4:**
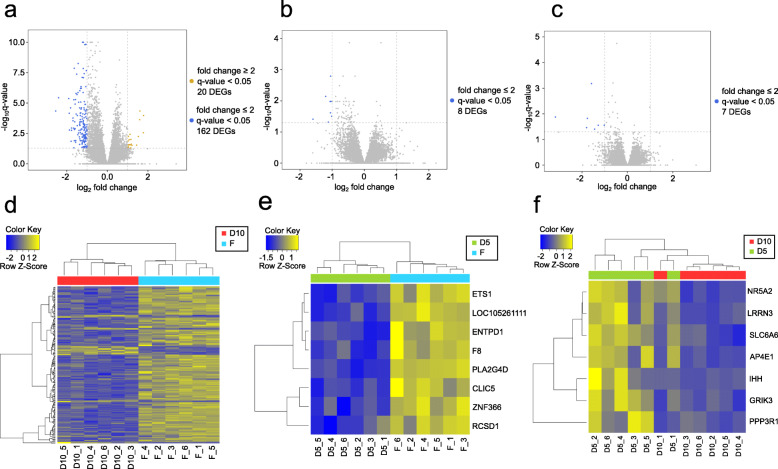
Influence of dehydration on the gene expression in cat ovarian cortex. **a** Volcano plot showing differentially expressed genes (DEGs) meeting the conditions of |fold change| ≥ 2 and q-value < 0.05 for comparison pairs D10 vs. F (dehydration for 10 min and rehydration compared to fresh tissue), **b** D5 vs. F (dehydration for 5 min compared to fresh tissue) and (**c**) D10 vs. D5 (dehydration for 10 min compared to dehydration for 5 min). Yellow dots represent up-regulated genes, blue dots represent down-regulated genes, and grey dots represent the genes that were not differentially expressed. Heatmap of the one-way Hierarchical Clustering Analysis (Euclidean Method, Complete Linkage) using Z-score for normalized value (log_2_ based) for comparison pairs (**d**) D10 vs. F, (**e**) D5 vs. F and (**f**) D10 vs. D5. Bottom of heatmap is labeled with sample IDs representing condition (D10, D5, F) and animal (1 to 6)

One hundred eighty two genes were differentially expressed in the ovarian cortex after dehydration for 10 min and rehydration (comparison pair D10 vs. F; Fig. 4a, Additional file 2). Of these, 20 were upregulated and included 5 protein coding genes, 1 pseudogene, 12 lncRNAs and 2 rRNAs. One hundred sixty two genes were downregulated and included 143 protein coding genes, 2 pseudogenes and 17 lncRNAs. Eight genes were differentially expressed in the ovarian cortex after dehydration for 5 min and rehydration (comparison pair D5 vs. F; Fig. 4b). All 8 DEGs were downregulated protein coding genes and 7 of them were also significantly downregulated in the comparison pair D10 vs. F and one gene (RCSD1) was downregulated in D10 vs. F below fold change cutoff of 2 (Additional file 3). Seven genes were differentially expressed in the ovarian cortex after dehydration for 10 min compared to dehydration for 5 min (comparison pair D10 vs. D5; Fig. 4c). All 7 DEGs were downregulated protein coding genes and 5 of them were also significantly downregulated in the comparison pair D10 vs. F (Additional file 2). The two DEGs that were not shared between comparison pairs D10 vs. D5 and D10 vs. F (IHH and PPP3R1) were non-significantly upregulated in the comparison pair D5 vs. F, explaining such difference (Additional file 3).

Hierarchical clustering divided DEGs from comparison pair D10 vs. F into two clusters (Fig. 4d), same for D5 vs. F (Fig. 4e). DEGs from comparison pair D10 vs. D5 could not be divided completely, indicating higher similarity between these two sets of DEGs (Fig. 4f).

**Fig. 5:**
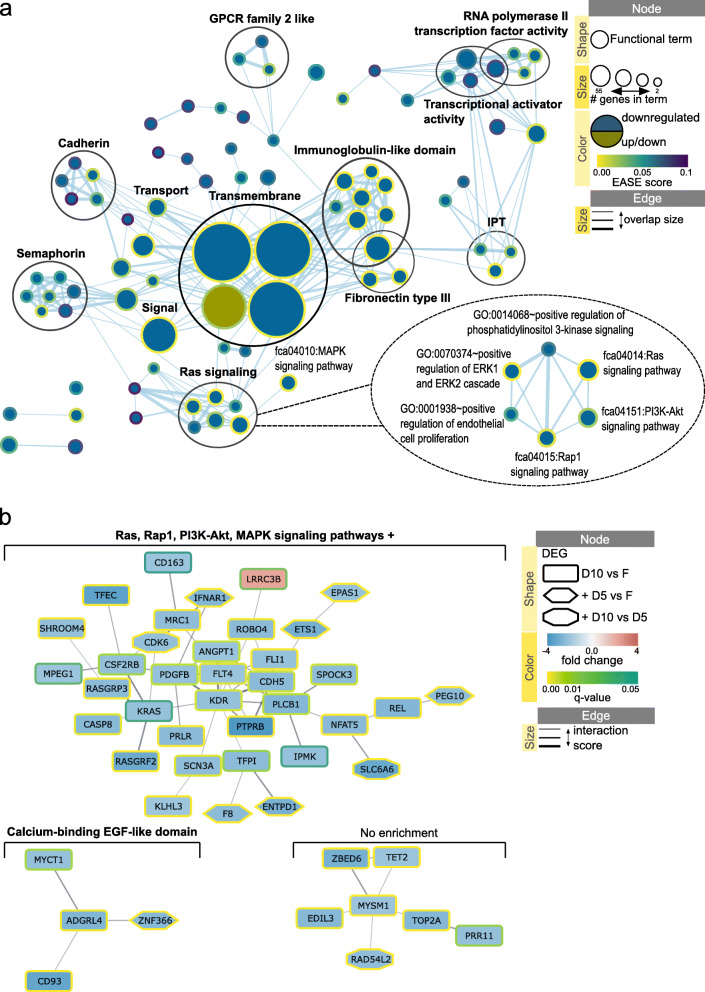
Network visualization of the effect of dehydration/rehydration on cat ovarian cortex. **a** Enrichment map displaying enriched gene-sets based on differentially expressed genes between dehydrated for 10 min (D10) and fresh (F) tissue. Blue node color represents enrichment in fresh tissue (i.e., down-regulation after 10 min dehydration), whereas green color represents enrichment in both dehydrated and fresh tissue (i.e., both up-regulation and down-regulation after 10 min dehydration). Borders are colored according to the enrichment *p*-value (EASE score). Clusters of functionally related gene-sets were identified using DAVID tool, manually circled and assigned a label. Additional file 6 contains the web session of the network for interactive view. **b** STRING network for 134 proteins retrieved from the list of differentially expressed genes in comparison pair D10 vs. F. Full gene list of the pair D10 vs. F resulted in 71 functional associations between 41 of the proteins; proteins with less than three associations to other proteins in the network were removed. Each cluster was analyzed for functional enrichment within STRING app. Nodes are shaped according to the comparison pair source of differentially expressed genes. Nodes are colored according to the gene abundance (fold change) in dehydrated/rehydrated compared to fresh cortex; borders are colored according to the adjusted *p*-value of the differentially expressed gene. The confidence cutoff was set to 0.4, the confidence score of each interaction is mapped to the edge thickness. Additional file 7 contains the web session of the network for interactive view

Out of 148 protein coding DEGs in comparison pair D10 vs. F, 147 were annotated in DAVID database; functional analysis was performed separately for upregulated (6) and downregulated (141) annotated DEGs. As in the section above, DAVID annotation clustering tool was used to create clusters of functionally related terms; information for each resulting annotation cluster is presented in Table 1. Figure 5a visualizes DAVID results for comparison pair D10 vs. F in an Enrichment Map network; Additional file 6 contains the web session of the network with interactive view and data table.

All DEGs in comparison pairs D5 vs. F (8) and D10 vs. D5 (7) were annotated in DAVID database, with one functional term enriched in D5 vs. F (‘fca04014:Ras signaling pathway’, EASE score 0.094) and two in D10 vs. D5 (‘Transport’, EASE score 0.014; ‘fca04724:Glutamatergic synapse’, EASE score 0.062).

STRING network of protein-protein interactions was created for comparison pair D10 vs. F based on 134 out of 148 protein coding DEGs present in domestic cat STRING database. For each cluster of genes that had at least three direct protein interactions, enrichment analysis via the STRING app was performed (Table 2). Figure 5b visualizes STRING results for comparison pair D10 vs. F in a STRING network. DEG data from comparison pairs D5 vs. F and D10 vs. D5 was mapped to the corresponding nodes. Additional file 7 contains the web session of the network with interactive view and data table.

DAVID functional analysis retrieved only one enriched term from 6 upregulated annotated DEGs, ‘GO:0016021~integral component of membrane’, which was part of Transmembrane cluster (Fig. 5a) and included 3 DEGs (ND3, LRRC3B and LOC101099262; Additional file 6). The same functional term was also enriched when analyzing downregulated DEGs and included 38 genes. The rest of 81 enriched terms were retrieved only from the list of downregulated DEGs (Fig. 5a).

The largest protein interaction cluster contained 36 genes with 35 of them downregulated and one (LRRC3B) upregulated (Fig. 5b). Functional enrichment for this cluster retrieved 74 enriched terms (Table 2), of which Ras, Rap1, PI3K-Akt and MAPK signaling KEGG pathways were highly enriched and included the highest number of genes from the cluster (Fig. 5b). These same KEGG terms were also highly enriched in DAVID analysis and formed Ras signaling cluster connected with ‘fca04010:MAPK signaling pathway’ term (Fig. 5a, Table 1). DEGs from comparison pairs of D5 vs. F (6 DEGs) and D10 vs. D5 (2 DEGs) were part of the ‘Ras, Rap1, PI3K-Akt, MAPK signaling pathways +’ protein interaction cluster (Fig. 5b). KEGG term of Ras signaling pathway was also the only enriched term for the comparison pair D5 vs. F, as presented above, and included ETS1 and PLA2G4D genes.

Collective data showed that changes in ovarian cortex transcriptome after the dehydration protocol measured within 30 min of rehydration included downregulation of genes mainly involved in Ras, Rap1, PI3K-Akt and MAPK signaling pathways, as well as RNA polymerase II transcription factor activity and other functional and protein domain terms summarized in Fig. 5a and Table 1.

### Ovarian cortex response to vitrification and dehydration involves different sets of differentially expressed genes, but shares some major functional terms

**Fig. 6:**
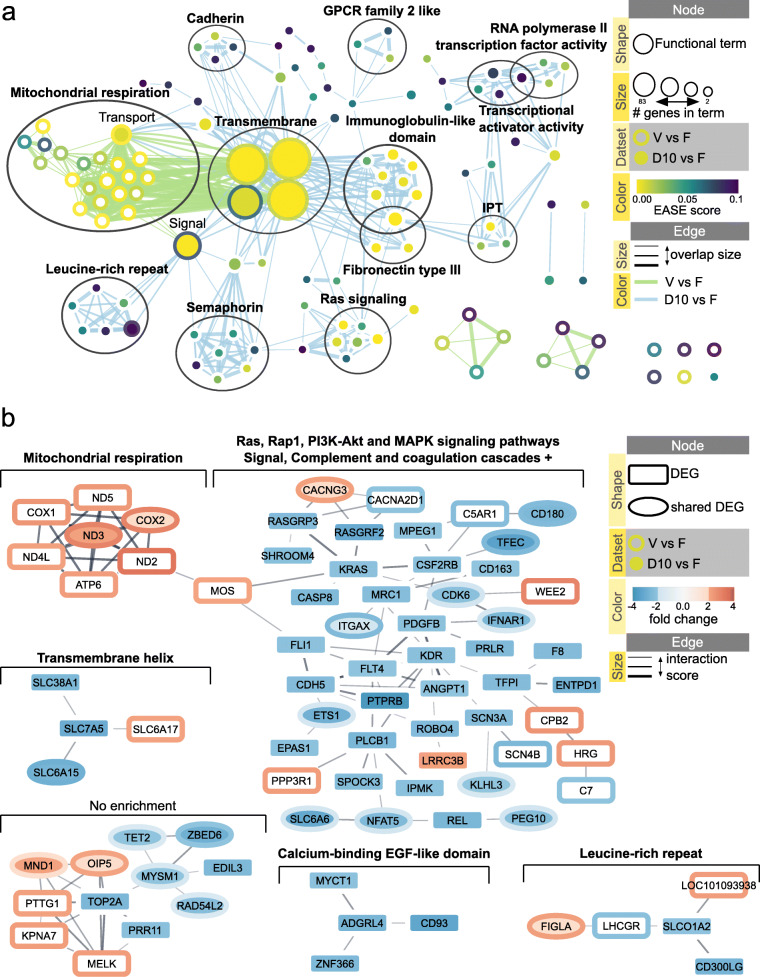
Comparison of the effects of vitrification and dehydration on the cat ovarian cortex. **a** Enrichment map displaying the enriched gene-sets based on differentially expressed genes between vitrified (V) and fresh (F) tissue and dehydrated for 10 min (D10) and fresh (F) tissue. Nodes are colored according to the enrichment *p*-value (EASE score) of D10 vs. F; borders are colored according to the enrichment *p*-value of V vs. F. Clusters of functionally related gene-sets were identified using DAVID tool, manually circled and assigned a label. Additional file 8 contains the web session of the network for interactive view. **b** STRING network for proteins retrieved from the list of differentially expressed genes in comparison pairs V vs. F and D10 vs. F; proteins with less than three associations to other proteins were removed for the network. Each cluster was analyzed for functional enrichment within STRING app. Nodes are shaped according to the gene list source. Nodes are colored according to the gene abundance (fold change) in comparison pair D10 vs. F; borders are colored according to the gene abundance (fold change) in comparison pair V vs. F. The confidence cutoff was set to 0.4, the confidence score of each interaction is mapped to the edge thickness. Additional file 9 contains the web session of the network for interactive view

To compare the transcriptomic response of ovarian cortex to vitrification and dehydration, 102 annotated DEGs from comparison pair V vs. F and 147 annotated DEGs from D10 vs. F were each analyzed for functional enrichment via DAVID and visualized using Enrichment Map network (Fig. 6a). Additional file 8 contains the web session of the network with interactive view and data table. Functional clustering revealed similar results as above with only two differences: (1) no enriched Transmembrane cluster for DEGs from V vs. F and (2) new cluster of Leucine-rich repeat for DEGs from D10 vs. F (Table 1). As seen on Fig. 6a, the terms enriched in comparison pairs V vs. F were clearly separated from the ones enriched in D10 vs. F, with only 7 enriched terms shared between the comparison pairs: ‘Membrane’, ‘Transmembrane’, ‘Transmembrane helix’ and ‘GO:0016021~integral component of membrane’ (all four are in Transmembrane cluster), ‘Transport’, ‘Signal’ and ‘IPR001611:Leucine-rich repeat’.

STRING network of protein interactions was build based on 214 DEGs derived from both V vs. F and D10 vs. F comparison pairs (Fig. 6b). Additional file 9 contains the web session of the network with interactive view and data table. DEG fold change data with q-value < 0.05 cutoff (but not fold change cutoff) from each comparison pair was mapped to the nodes of the network for better visualization of shared genes (Fig. 6b). One big protein interaction cluster was formed from 55 genes, of which 14 genes were differentially expressed only in comparison pair V vs. F (one gene PPP3R1 was also differentially expressed in D10 vs. D5), 28 genes were differentially expressed only in comparison pairs D10 vs. F, D5 vs. F and D10 vs. D5, and 13 genes were differentially expressed (with no fold change cutoff) in both V vs. F and D10 vs. F (data table in Additional file 9). This big cluster could be divided into two smaller ones with retrieved functional enrichments of Mitochondrial respiration (mainly DEGs from V vs. F) and Ras, Rap1, PI3K-Akt and MAPK signaling pathways + (mainly DEGs from D10 vs. F; Fig. 6b). This second Ras signaling cluster also included DEGs from V vs. F that formed their own protein interaction cluster in STRING network of Fig. 3b, therefore adding terms ‘Signal’ and ‘Complement and coagulation cascades’ to the cluster (Table 2).

Protein interaction clusters of Transmembrane helix and Leucine-rich repeat contained DEGs from both comparison pairs V vs. F and D10 vs. F, similar to the results obtained with DAVID analysis (Fig. 6a, b).

**Fig. 7:**
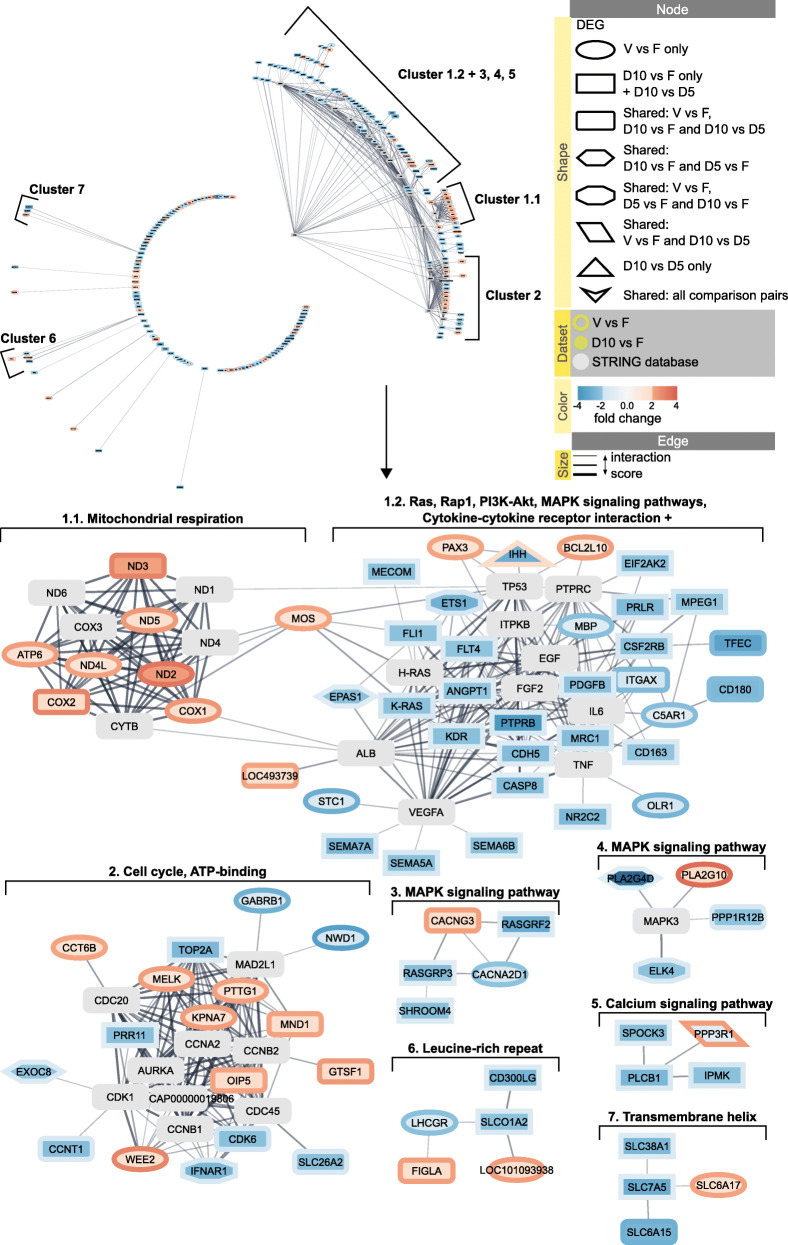
Summarized overview using STRING network of differentially expressed genes from all comparison pairs. The network was built using 25 additional directly interacting proteins derived from STRING domestic cat database. Overview is presented in radial layout. MCL algorithm was used for clustering based on interaction score, with granularity set to 2 and edge cutoff to 0.4. Only clusters with genes that have more than three protein associations are presented. Each cluster was analyzed for functional enrichment within STRING app. Nodes are shaped according to the comparison pair source of differentially expressed genes. Nodes are colored according to the gene abundance (fold change) in comparison pair D10 vs. F; borders are colored according to the gene abundance (fold change) in comparison pair V vs. F. The confidence cutoff was set to 0.4, the confidence score of each interaction is mapped to the edge thickness. Additional file 10 contains the web session of the network for interactive view

Finally, it was possible to integrate all 241 out of 253 protein coding DEGs plus 25 additional directly interacting proteins derived from STRING domestic cat database into one big STRING network (Fig. 7, Additional file 10). The DEG data was mapped from all four comparison groups to the network for a summarized visualization; Additional file 3 contains united DEG data. The 25 database derived proteins connected additional genes to the main protein interaction cluster, which resulted now in 132 genes (107 DEGs and 25 database proteins). MCL clustering algorithm in clusterMaker app was used to divide this cluster into smaller protein interaction clusters and analyzed functional enrichment in clusters with at least three protein interactions (Fig. 7). As before, mitochondrial respiration cluster consisting of DEGs from comparison pair V vs. F. (Fig. 7) was identified. The Ras signaling cluster that was observed previously this time could be further divided into 4 more clusters: cluster 1.2, Ras, Rap1, PI3K-Akt, MAPK signaling pathways, Cytokine-cytokine receptor interaction +; cluster 3 and 4, MAPK signaling pathway; cluster 5, Calcium signaling pathway (Fig. 7). Protein interaction clusters from V vs. F and D10 vs. F that previously did not return any enrichment this time were connected by additional database proteins to form a cluster enriched in Cell cycle and ATP-binding (Fig. 7).

All genes had the same up- or downregulation trend for both V vs. F and D10 vs. F, except for PPP3R1, IHH, GRIK3 and IGFN1 (Additional file 3, Additional file 10). PPP3R1 was significantly upregulated in comparison pair V vs. F and non-significantly upregulated in D5 vs. F, but significantly downregulated in comparison pair D10 vs. D5, with a downregulation trend in D10 vs. F (Additional file 3). IHH and GRIK3 were downregulated in comparison pairs D10 vs. D5 and D10 vs. F, but had an upregulation trend in D5 vs. F and V vs. F. IGFN1 was significantly downregulated in V vs. F and non-significantly in D5 vs. F, but had an upregulation trend in D10 vs. F and D10 vs. D5.

In sum, vitrification and dehydration protocols mainly changed the expression of different genes in the ovarian cortex that result in different enriched functional terms; however, some of these genes were united by protein-protein interactions in silico and formed one major cluster of mitochondrial respiration and Ras/MAPK signaling pathways.

## Discussion

Collective results showed, for the first time, the immediate effects of vitrification and microwave-assisted dehydration on the global transcriptome dynamics of domestic cat ovarian cortex. Differences in transcriptomic response to vitrification/warming or dehydration/rehydration were observed. Vitrification and warming mainly upregulated the expression of mitochondrial (mt) DNA genes that play key role in mitochondrial respiration, while dehydration for 10 min and rehydration exhibited major downregulation of gene expression, particularly of genes enriched in Ras, Rap1, PI3K-Akt and MAPK signaling pathways and RNA Polymerase II (Pol II) transcription regulation. Dehydration for 5 min had almost negligible effect on the gene expression, only downregulating eight genes, including transcription factor ETS1 and phospholipase PLA2G4D enriched in Ras signaling pathway. Comparison of dehydration for 10 min with 5 min showed downregulation of only seven genes.

A recent study using RNA-seq to measure the response of mouse ovarian tissue to vitrification followed by 20 days of auto-transplantation was reported [29]. Additionally, several studies used RNA-seq in recent years to measure global transcriptomic changes in oocytes after vitrification followed by culturing or in vitro maturation [24–28]. However, these earlier studies did not analyze the immediate transcriptomic changes after vitrification or rehydration, and, therefore, are difficult to compare with ours.

### Effect of vitrification

Previously, we showed that ovarian tissue vitrification used in this study did not significantly alter the percentage of morphologically normal follicles observed in the cat cortex, and only mildly lowered the percentage of viable follicles and RNA synthesis [8]. Our current transcriptomic data shows no significant upregulation of proapoptotic factors and no enrichment of downregulated genes in RNA synthesis process, supporting these previous observations. The biggest effect of the vitrification protocol in the present study was measurable within 20 min post-warming and involved upregulation of the expression of mtDNA genes related to mitochondrial respiration. These mtDNA genes form different respiratory chain complexes and include ND2, ND3, ND4L and ND5 (complex I), COX1 and COX2 (complex IV), and ATP6 (complex V). Previous studies on the cat ovarian tissue vitrification showed no changes in the integrity of mitochondria based on transmission electron microscopy analysis [34]. In the meantime, many reports show adverse effect of vitrification on oocyte mitochondrial function [35–38].

Mitochondria have diverse activities in the regulation of cellular homeostasis, including ATP production, control of calcium homeostasis, lipid metabolism, apoptosis and redox regulation [39]. Out of these, energy conversion and production of ATP is the most critical vital cellular activity of mitochondria that involves electron transfer complexes I to V [40]. Recent study showed that activation of mitochondrial respiration, linked to improved efficiency of the electron transport chain and blocking reactive oxygen species accumulation, is a critical component of an adaptive endoplasmic reticulum (ER) stress response, requiring calcium signaling via calcineurin [41]. During ER stress, Ca2+ is released from the ER and enters mitochondria; this process is suggested to regulate oxidative phosphorylation and initially represent an adaptive response to acute ER stress [42, 43]. In our study, expression of seven mtDNA genes involved in mitochondrial respiration as well as gene expression of calcineurin (PPP3R1) were increased immediately after warming of ovarian tissue, potentially indicating on the possibility of the ER stress and activation of mechanisms to alleviate it during warming.

### Effect of dehydration

Using the same microwave-assisted dehydration protocol, our laboratory recently showed that dehydration for 5 or 10 min did not significantly alter the percentage of morphologically normal follicles and did not increase DNA damage [18]. In the current study we did not identify significant upregulation of gene expression of proapoptotic factors after tissue rehydration, supporting that observation. Previously we also showed that dehydration for 10 min significantly inhibited the RNA synthesis as measured by incorporation of the uridine analog into transcripts produced by RNA Pol I and II, while dehydration for 5 min showed no changes in RNA synthesis [18, 44]. Similarly, our current transcriptomic data shows major downregulation of genes, including genes with enrichment in RNA Pol II transcription factor activity, after 10 but not 5 min of dehydration.

In the last few decades, studies investigating molecular and physiological changes in cells after exposure to stress provided invaluable models for elucidating mechanisms of key cellular processes and showed conserved massive transcription downregulation caused by different types of stresses [30]. Studies showed that components of the translation machinery are transcriptionally repressed within 10 min of heat or celastrol stress exposures, while RNA-processing complexes have a more delayed decline; and such response seems to be conserved from fly to human [30]. A study on the effect of celastrol on the global transcription in human cells revealed two major waves of nascent RNA response, one within 10 min, and a second 40–60 min after treatment, and showed that down-regulated genes specific to heat shock response are enriched in MAPK signaling and cell-cycle progression, whereas down-regulated genes that were shared in heat and celastrol responses were enriched for ribosomal formation and translation [45]. Our transcriptomic data shows massive downregulation of genes within 30 min of rehydration after 10 min of dehydration, with a significant set of genes enriched in MAPK signaling pathway. Cells have a complex sensing network to monitor cellular stress and change ribosomal DNA transcription accordingly via critical controllers such as PI3K/AKT/mTORC1, RAS/RAF/ERK pathways and MYC transcription factor [46]. In the present study, genes with enrichment in PI3K-Akt, Ras and ERK pathways, as well as MYC target gene MYCT1, were all downregulated after 10 min of dehydration. At the same time, the only enriched functional term after 5 min dehydration was Ras signaling pathway and no massive transcription downregulation was observed. Based on our transcriptomic data and studied literature, we hypothesize that 10 min of dehydration causes significant stress to ovarian tissue that is already measurable within 30 min of warming and includes downregulation of signaling pathways and MYCT1 that are potentially linked to massive downregulation of gene expression, while 5 min of dehydration causes only mild stress with initial moderate inhibition of Ras signaling that doesn’t lead to massive transcript downregulation. It is important to note that 5 min of dehydration still exhibits a downregulation trend due to small gene expression differences between 10 and 5 min of dehydration.

### Vitrification vs. dehydration stress

Cells encounter external and internal stress conditions that can disrupt homeostasis and include elevated temperatures, toxins, hypoxia, cancer and ageing, each of which compromises cellular functions by affecting proteins, membranes and DNA [47]. To defend themselves from these adverse conditions, cells activate rapid and transient programs that adjust RNA and protein synthesis, cytoskeletal and membrane integrity and the metabolic state of the cell [30]. Extensive studies on cellular stress responses showed strikingly similar patterns of transcriptional changes after two diverse stressors, heat and celastrol [45, 48, 49]. These studies indicated that heat and celastrol stresses activate same pathways to increase expression of chaperones but have different effect on cellular compartments, such as the ER. In the present study we observed different transcriptomic response of the ovarian tissue to stresses caused by vitrification and 10 min dehydration. At the same time, transcriptomic response to 5 min dehydration was mild and was partly similar to 10 min dehydration effect (initial inhibition of Ras signaling, downregulation trend) and partly to vitrification effect (same trend in expression of four genes with opposing to 10 min dehydration response, PPP3R1, IHH, GRIK3 and IGFN1). We hypothesize that the extent of stress differs between all these conditions: (1) vitrification mild stress activates ER stress defense response increasing mitochondrial respiration activity and gene expression of calcineurin PPP3R1; (2) 5 min dehydration stress does not inhibit calcineurin PPP3R1 expression, leaving possibility for defensive stress response similar to vitrification, but at the same time downregulates genes enriched in Ras signaling pathway; (3) the stronger stress of 10 min dehydration already significantly downregulates PPP3R1 compared to both fresh tissue and tissue after 5 min dehydration, does not upregulate any known protective factors and massively inhibits transcription and associated signaling pathways.

Our recent study on oocytes’ germinal vesicles in domestic cat showed similar effect of vitrification and dehydration protocols on epigenetic patterns but indicated less structural damage after dehydration and rehydration compared to vitrification and warming [22]. Opposite to that, in our current study, ovarian tissue response to dehydration/rehydration seems to be more intense and potentially damaging to cells compared to vitrification/warming. This difference may potentially be explained by the absence of cytoplasmic organelles and cell-cell communications in the experiment on germinal vesicle. This could lead to the absence of any defense mechanisms activation during vitrification and warming and/or no inhibition of signaling that would lead to massive transcription inhibition during dehydration and rehydration.

### Study limitations and next steps

We measured global transcriptomic changes only within 30 min of reanimation after preservation, and it is very likely that these observed effects might change with time and can become more detrimental with possible activation of apoptotic processes. It is therefore extremely important to optimize the steps after reanimation to return the ovarian tissue to its normal functional and structural state. We showed before that culturing bovine ovarian tissue after vitrification and warming from three to 7 days improves follicular integrity [50]. Additionally, a study on human oocytes at germinal vesicle stage showed a transient increase in mitochondrial membrane potential after vitrification that returned to normal after 2 days of culture [51]. Therefore, in future we should focus on optimization of culture protocol of ovarian tissue after reanimation. One of such optimization steps may include the addition of epidermal growth factor during culture, as our previous study showed its beneficial effect in prepubertal cat ovary by enhancing stromal cell proliferation via MAPK and PI3K pathways [52]. We observed significant downregulation of genes involved in MAPK and PI3K pathways after 10 min of dehydration, and culturing rehydrated ovarian tissues with addition of epidermal growth factor may improve it. Similarly, we can use the transcriptomic data and functional networks presented here to explore additional steps of preservation protocol optimization. For example, dehydration for 10 min led to decrease in the expression of genes enriched in functional terms related to cadherins, cell-cell adhesion factors that play important roles in membrane fusion. Such disturbance in cell-cell adhesion and communication may be a result of exposure to the high osmotic solution during preservation process [53], and a recent study has shown that the addition of collagen before the exposure to vitrification solution improves viability of secondary follicles in the ovarian tissue [54].

It is important to note that our study was performed on ovarian tissues from prepubertal cats and therefore our data can only be applied to ovarian cortex with high number of primordial and primary follicles, and low number of large follicles. We may see a different effect of vitrification and dehydration protocols on the ovarian tissue with high number of large follicles, as such follicles are more susceptible to cryoinjury. Additionally, we measured transcriptomic changes in the whole ovarian tissue and, therefore, could not distinguish specific response based on the cell type. We did not, however, identified any significant changes in the gene expression of known cellular markers reported in the ovary [32, 33]. Finally, there are many levels at which cellular response to stress can be modulated, and global transcription of mRNA is only one of them; we acknowledge that our study was limited to the measurement of an initial effect on the transcription of mature mRNA. Future studies should focus on ovarian localization of differentially expressed genes with proposed biological significance, as well as changes in their protein levels in response to vitrification and dehydration protocols.

## Conclusions

We showed, for the first time, different effects of vitrification and microwave-assisted dehydration protocols on the global transcriptome of the ovarian cortex (using domestic cat as a biomedical model).

The acquired transcriptomic data and the map of affected functional networks bring us closer to deciphering the complex response of ovarian tissue to non-physiological stresses and provide directions for future studies and improvements of preservation protocols that can be used in biomedicine and conservation efforts of endangered species.

## Methods

### Collection of ovarian tissues

Ovaries from prepubertal (3–6 months, as determined by medical records) domestic cats were collected at local veterinary clinics as byproducts from owner-requested routine ovariohysterectomies and transported at 4 °C to the laboratory within 6 h of excision. Ovarian cortical tissues were dissected into 1 × 1 × 0.2 mm pieces in dissection medium (minimum essential medium [MEM with Hank’s salt; Gibco Laboratories, Gaithersburg, MD] supplemented with 10 mM HEPES, 1 mM pyruvate, 2 mM L-glutamine, 100 IU.ml penicillin, 100 μg/ml streptomycin and 0.1% bovine serum albumin) [8]. For each animal, cortical pieces were either stabilized immediately in RNAlater™ solution (Invitrogen, Carlsbad, CA) or processed for vitrification or dehydration as described below. All chemicals and reagents were purchased from Sigma-Aldrich (St. Louis, MO), unless otherwise indicated.

### Vitrification and warming

Vitrification was performed using needle immersion vitrification protocol reported previously for domestic cat ovarian cortex [8]. Cortical pieces were threaded onto a 30-G needle (six pieces per needle; BD PrecisionGlide needle, Thermo Fisher Scientific, Waltham, MA) with space in between, exposed to equilibration solution (7.5% dimethyl sulfoxide (DMSO) + 7.5% ethylene glycol (EG) + 20% fetal bovine serum (FBS) in base medium) for 10 min at 4 °C followed by a vitrification solution (15% DMSO + 15% EG + 20% FBS in base medium) for 10 min at 4 °C, then plunged directly into liquid nitrogen and stored for at least 48 h in liquid nitrogen. Warming was performed by quickly transferring the needles to a sucrose gradient (1, 0.5, 0.25 and 0 M in base medium) for 5 min at each step at 37 °C. Cortical pieces were then removed from the needles and incubated in RNAlater™ stabilization solution overnight at 4 °C. After removal of stabilization solution, tissues were stored at − 80 °C until RNA isolation.

### Microwave-assisted dehydration and rehydration

Dehydration was performed using microwave assisted protocol reported previously for domestic cat ovarian cortex [18]. Cortical pieces from the same ovaries used in vitrification were threaded onto a 30-G needle as in vitrification step, then immersed in 10 μg/ml digitonin for 3 min to permeabilize the cells. After rinsing in dissection medium, cortical pieces were exposed to 1 M trehalose for 10 min. Excess trehalose solution was gently dabbed off on wipes before moving cortical pieces from the needle onto conjugate-release glass fiber filters (Whatman, Maidstone, UK). Samples were dehydrated in a SAM 255 microwave (CEM, Matthews, NC) for 5 or 10 min at 20% power (about 100 W power output) with upper temperature threshold set at 40 °C. Dry cortical pieces on filters were immediately rehydrated in dissection medium for 30 min at room temperature and then incubated in RNAlater™ stabilization solution overnight at 4 °C. After removal of stabilization solution, tissues were stored at − 80 °C until RNA isolation.

### RNA preparation

Total RNA was isolated from up to 10 mg of tissue using PureLink™ RNA Mini Kit with on-column DNase Set (Invitrogen); tissue was homogenized in RNA lysis buffer using TissueLyser (Qiagen, Hilden, Germany; 2 × 2 min at 30 Hz; 5 mm stainless steel beads). Concentration and purity of isolated RNA was measured with NanoDrop™ spectrophotometer (Thermo Fisher Scientific); RNA integrity was assessed using 2100 Bioanalyzer instrument (Agilent Technologies, Santa Clara, CA). Purified RNA was stored in nuclease-free water at − 80 °C until library preparation.

### Library preparation and transcriptome sequencing

Only samples with RIN ≥ 7 were used for library preparation. Sequencing libraries were generated using TruSeq Stranded mRNA LT Sample Prep Kit from Illumina (San Diego, CA) according to the manufacturer’s recommendations. In short, the workflow included randomly fragmenting total RNA for short read sequencing, reverse transcribing fragmented RNA into cDNA, ligating adaptors onto both ends of the cDNA fragments, amplifying cDNA and selecting fragments with insert sizes between 200 and 400 bp. The libraries were sequenced 150 bp paired-end using an Illumina NovaSeq 6000 System at the Psomagen Inc. (formerly Macrogen Corp., Rockville, MD) with 30 million reads depth per sample and 6 biological replicates per group.

### Quality control

The quality of produced data was determined by the phred quality score at each cycle using FastQC (v. 0.11.7). Trimmomatic (v. 0.38) [55] program was used to remove adapter sequences and bases with base quality lower than three from the ends. Using sliding window method, bases of reads that did not qualify for window size 4 and mean quality 15 were trimmed. Afterwards, reads with length shorter than 36 bp were dropped to produce trimmed data. Quality information for each sample after trimming is provided in Additional file 11: Table S1.

### Reads mapping and gene expression levels quantification

Trimmed reads were mapped to reference genome GCF_000181335.3_Felis_catus_9.0 with HISAT2 (v. 2.1.0) [56], which is known to handle spliced read mapping through Bowtie2 (v. 2.3.4.1) aligner, splice-aware aligner. Additional file 12: Table S2 shows the statistic obtained from HISAT2.

After the read mapping, known genes and transcripts were assembled with StringTie (v. 1.3.4d) [57, 58] based on reference genome model. After assembly, the abundance of gene/transcript was calculated in the read count and normalized value as FPKM (Fragment per Kilobase of transcript per Million mapped reads) for a sample.

### Differential expression analysis

For 24 samples, if more than one read count value was 0, it was not included in the analysis. Therefore, from total of 35,543 genes, 17,767 were excluded and only 17.776 genes were used for statistical analysis. In order to reduce systematic bias, we estimated the size factors from the count data and applied Relative Log Expression (RLE) normalization with DESeq2 R library [59]. In case of DESeq2, read count+ 1 & Logarithm value was used to visualize the plots before normalization, and regularized log transformed value was used to visualize the plots after normalization.

DEG (Differentially Expressed Genes) analysis was performed on all data using the DESeq2 R package with 4 pairwise comparisons: V vs. F, D10 vs. F, D5 vs. F and D10 vs. D5. DESeq2 provides statistical routines for determining differential expression in digital gene expression data by using a model based on a negative binominal distribution [59]. The resulting *P*-values were adjusted using Benjamini and Hochberg’s approach for controlling the false discovery rate. Genes with absolute fold change |fc| ≥ 2 and an adjusted *P-*value (q-value) < 0.05 as calculated by DESeq2 were considered differentially expressed.

### Functional enrichment analysis using DAVID and enrichment map

The acquired DEG list was analyzed for gene-set enrichment with DAVID tool [60] using functional annotation databases of Gene Ontology, KEGG Pathways, InterPro Domains, UniProt Keywords and SMART domains, setting species to domestic cat. For each comparison pair, total DEGs and separately up- and downregulated DEGs were analyzed. Clustering function of DAVID was used to unite similar annotation terms. EASE score (modified Fisher Exact *p*-value of enrichment) was set to 0.1. Functional enrichment network was built based on DAVID output charts of gene-set enrichment for each comparison pair using Enrichment Map app (v. 3.3.0, [61]) in Cytoscape software (v. 3.8.0, [62, 63]) with overlap parameter set to 0.5. YFiles Layout Algorithms app (v. 1.1) was used to help visualize networks.

### In silico protein-protein interaction analysis using STRING

In silico protein-protein interaction analysis of DEGs was performed on the basis of the STRING database for the domestic cat [64, 65]. Interaction network was built based on the list of DEGs for each comparison pair using stringApp (v. 1.5.1, [66]) in Cytoscape with confidence cutoff score set to 0.4 and maximum additional interactors set to 0 (25 in network of Fig. 7, Additional file 10). Clustering was performed using MCL cluster mode in clusterMaker 2 app (v. 1.3.1, [67]) with granularity parameter (inflation value) set to 2, array source set to stringdb::score, edge weight cutoff set to 0.4. Functional enrichment was performed using domestic can genome as a background, enriched terms were analyzed with varying redundancy cutoff settings.

## Supplementary Information

**Additional file 1.** Differentially Expressed Genes (DEGs) in vitrified (V) compared to fresh (F) ovarian tissue using a cutoff of |fold change| ≥ 2 and q-value < 0.05.

**Additional file 2.** Differentially Expressed Genes (DEGs) in dehydrated for 10 min (D10) compared to fresh (F) ovarian tissue (Sheet-1), dehydrated for 5 min (D10) compared to F (Sheet-2) and D10 compared to D5 (Sheet-3) using a cutoff of |fold change| ≥ 2 and q-value < 0.05.

**Additional file 3.** Differentially expressed genes (DEGs) from at least one comparison pair using cutoff of |fold change| ≥ 2 and q-value < 0.05 with corresponding DEG analysis data from the rest of comparison groups.

**Additional file 4.** Web session interactive view with data table of Enrichment Map network for comparison pair V vs. F. For description and legend refer to Fig. 3a.

**Additional file 5.** Web session interactive view with data table of STRING network for comparison pair V vs. F. For description and legend refer to Fig. 3b.

**Additional file 6.** Web session interactive view with data table of Enrichment Map network for comparison pair D10 vs. F. For description and legend refer to Fig. 5a.

**Additional file 7.** Web session interactive view with data table of STRING network for comparison pair D10 vs. F (tissue dehydrated for 10 min and rehydrated compared to fresh) with mapped data from comparison pairs D5 vs. F and D10 vs. D5. For description and legend refer to Fig. 5b.

**Additional file 8.** Web session interactive view with data table of Enrichment Map network for comparison pairs V vs. F and D10 vs. F. For description and legend refer to Fig. 6a.

**Additional file 9.** Web session interactive view with data table of STRING network for comparison pairs V vs. F and D10 vs. F. For description and legend refer to Fig. 6b.

**Additional file 10.** Web session interactive view with data table of STRING network for all comparison pairs with 25 additional database derived proteins. For description and legend refer to Fig. 7.

**Additional file 11: Table S1.** Trimmed data statistics.

**Additional file 12:** **Table S2.** Mapped data statistics.

## Data Availability

The sequence data generated and analyzed during the current study is available in the NCBI SRA repository, BioProject PRJNA662384. The differential gene expression datasets supporting the conclusions of this article are included within the article as additional files.
