# Supplementary material for: Initial response of ovarian tissue transcriptome to vitrification or microwave-assisted dehydration in the domestic cat model
Source: BMC Genomics. 2020 Nov 25;21:828. doi: 10.1186/s12864-020-07236-z (PMC7690003; doi:10.1186/s12864-020-07236-z)
Supplement: Supplementary file 11 — Additional file 11: Table S1. Trimmed data statistics. [file 12864_2020_7236_MOESM11_ESM.docx]

Table S1. Trimmed data statistics.

| Sample ID | Total read bases | Total reads | GC(%) | Q20(%) | Q30(%) |
| --- | --- | --- | --- | --- | --- |
| CatOv-F-1 | 5,156,771,692 | 38,236,930 | 49.95 | 98.14 | 93.97 |
| CatOv-F-2 | 6,512,853,730 | 47,673,410 | 50.15 | 98.31 | 94.47 |
| CatOv-F-3 | 4,336,052,008 | 31,703,572 | 49.3 | 98.17 | 94.02 |
| CatOv-F-4 | 4,466,398,770 | 34,362,390 | 53.83 | 98.66 | 95.32 |
| CatOv-F-5 | 5,446,061,511 | 39,830,598 | 49.76 | 98.68 | 95.43 |
| CatOv-F-6 | 5,486,959,271 | 39,359,230 | 49.65 | 98.84 | 95.93 |
| CatOv-V-1 | 6,073,957,149 | 45,457,664 | 50.24 | 98.23 | 94.28 |
| CatOv-V-2 | 5,466,859,240 | 39,769,876 | 50.04 | 98.22 | 94.15 |
| CatOv-V-3 | 4,684,674,999 | 34,324,946 | 49.47 | 98.21 | 94.12 |
| CatOv-V-4 | 5,153,715,087 | 37,034,732 | 48.95 | 98.61 | 95.13 |
| CatOv-V-5 | 6,320,484,257 | 45,606,196 | 50.55 | 98.82 | 95.89 |
| CatOv-V-6 | 4,860,680,212 | 34,984,514 | 50.04 | 98.83 | 95.88 |
| CatOv-D5-1 | 5,536,792,760 | 40,328,824 | 49.39 | 98.24 | 94.19 |
| CatOv-D5-2 | 4,353,892,331 | 32,164,700 | 50.05 | 98.23 | 94.2 |
| CatOv-D5-3 | 4,373,202,686 | 32,230,352 | 49.72 | 98.16 | 94.03 |
| CatOv-D5-4 | 5,218,503,013 | 37,642,822 | 49.01 | 98.27 | 94.33 |
| CatOv-D5-5 | 5,501,069,047 | 39,942,498 | 49.64 | 98.82 | 95.87 |
| CatOv-D5-6 | 5,637,598,613 | 40,436,058 | 49.68 | 98.83 | 95.89 |
| CatOv-D10-1 | 4,361,400,339 | 32,015,606 | 49.83 | 98.17 | 94.04 |
| CatOv-D10-2 | 4,637,169,492 | 34,195,866 | 50.27 | 98.09 | 93.86 |
| CatOv-D10-3 | 4,225,594,961 | 31,160,556 | 50.07 | 97.99 | 93.63 |
| CatOv-D10-4 | 5,016,569,649 | 36,446,520 | 49.86 | 98.49 | 94.85 |
| CatOv-D10-5 | 5,291,730,434 | 38,747,484 | 51.09 | 98.8 | 95.85 |
| CatOv-D10-6 | 5,050,778,068 | 36,151,574 | 50.28 | 98.73 | 95.63 |

Sample IDs correspond to IDs in BioProject PRJNA662384, NCBI SRA

Total read bases: Total number of read bases after trimming

Total reads: Total number of reads after trimming

GC (%): GC Content

Q20 (%): Ratio of bases that have phred quality score greater than or equal to 20

Q30 (%): Ration of bases that have phred quality score greater than or equal to 30
