# Supplementary material for: Initial response of ovarian tissue transcriptome to vitrification or microwave-assisted dehydration in the domestic cat model
Source: BMC Genomics. 2020 Nov 25;21:828. doi: 10.1186/s12864-020-07236-z (PMC7690003; doi:10.1186/s12864-020-07236-z)
Supplement: Supplementary file 12 — Additional file 12: Table S2. Mapped data statistics. [file 12864_2020_7236_MOESM12_ESM.docx]

**Table S2. Mapped data statistics.**

| **Sample ID** | **# of processed reads** | **# of mapped reads (%)** | **% of mapped reads** | **# of unmapped reads** | **% of unmapped reads** |
| --- | --- | --- | --- | --- | --- |
| CatOv-F-1 | 38,236,930 | 37,400,658 | 97.81 | 836,272 | 2.19 |
| CatOv-F-2 | 47,673,410 | 46,700,770 | 97.96 | 972,640 | 2.04 |
| CatOv-F-3 | 31,703,572 | 31,059,499 | 97.97 | 644,073 | 2.03 |
| CatOv-F-4 | 34,362,390 | 33,727,125 | 98.15 | 635,265 | 1.85 |
| CatOv-F-5 | 39,830,598 | 39,074,784 | 98.10 | 755,814 | 1.90 |
| CatOv-F-6 | 39,359,230 | 38,657,629 | 98.22 | 701,601 | 1.78 |
| CatOv-V-1 | 45,457,664 | 44,583,702 | 98.08 | 873,962 | 1.92 |
| CatOv-V-2 | 39,769,876 | 39,071,941 | 98.25 | 697,935 | 1.75 |
| CatOv-V-3 | 34,324,946 | 33,701,842 | 98.18 | 623,104 | 1.82 |
| CatOv-V-4 | 37,034,732 | 36,433,279 | 98.38 | 601,453 | 1.62 |
| CatOv-V-5 | 45,606,196 | 44,842,438 | 98.33 | 763,758 | 1.67 |
| CatOv-V-6 | 34,984,514 | 34,414,648 | 98.37 | 569,866 | 1.63 |
| CatOv-D5-1 | 40,328,824 | 39,549,519 | 98.07 | 779,305 | 1.93 |
| CatOv-D5-2 | 32,164,700 | 31,546,531 | 98.08 | 618,169 | 1.92 |
| CatOv-D5-3 | 32,230,352 | 31,554,439 | 97.90 | 675,913 | 2.10 |
| CatOv-D5-4 | 37,642,822 | 35,661,527 | 94.74 | 1,981,295 | 5.26 |
| CatOv-D5-5 | 39,942,498 | 39,188,800 | 98.11 | 753,698 | 1.89 |
| CatOv-D5-6 | 40,436,058 | 39,736,312 | 98.27 | 699,746 | 1.73 |
| CatOv-D10-1 | 32,015,606 | 31,399,791 | 98.08 | 615,815 | 1.92 |
| CatOv-D10-2 | 34,195,866 | 33,481,334 | 97.91 | 714,532 | 2.09 |
| CatOv-D10-3 | 31,160,556 | 30,488,321 | 97.84 | 672,235 | 2.16 |
| CatOv-D10-4 | 36,446,520 | 35,781,704 | 98.18 | 664,816 | 1.82 |
| CatOv-D10-5 | 38,747,484 | 38,126,019 | 98.40 | 621,465 | 1.60 |
| CatOv-D10-6 | 36,151,574 | 35,494,404 | 98.18 | 657,170 | 1.82 |

Sample IDs correspond to IDs in BioProject PRJNA662384, NCBI SRA

Processed reads: Number of cleaned reads after trimming

Mapped reads: Number of reads mapped to reference

Unmapped reads: Number of reads that failed to align
